# Editorial to Special Issue “Theme Issue Honoring Prof. Dr. Ludger Wessjohann’s 60th Birthday: Natural Products in Modern Drug Discovery”

**DOI:** 10.3390/ijms23105835

**Published:** 2022-05-23

**Authors:** Hidayat Hussain

**Affiliations:** Department of Bioorganic Chemistry, Leibniz Institute of Plant Biochemistry, Weinberg 3, 06120 Halle (Saale), Germany; hidayat.hussain@ipb-halle.de

Nature continuously produces biologically useful molecules and provides mankind with life-saving drugs or therapies. Natural products (NPs) offer a vast, unique and fascinating chemical diversity, and these molecules have evolved for optimal interactions with biological macromolecules. Moreover, natural products feature pharmacologically active pharmacophores, which are pharmaceutically validated starting points for the development of new lead compounds. Over half of all approved (from 1981 to 2014) small-molecule drugs derived from NPs, including unaltered NPs, NPs synthetic derivatives and synthetic natural mimics, originated from an NP’s pharmacophore or template. According to the FDA, NPs and their derivatives represent over one-third of all FDA-approved new drugs, in particular for anticancer/antibiotic lead compounds, which are remarkably enriched with NPs.

Many scientists have contributed to this Special Issue (SI), which includes 16 papers, original articles along with review articles that give the readers of the *International Journal of Molecular Sciences* an updated and new perspective about natural products in drug discovery.

Multicomponent reactions (MCRs) are reactions in which three or more reagents are allowed to undergo a reaction in a one-pot fashion in which almost all the atoms from the starting materials are incorporated into the final product. Furthermore, these MCRs feature various advantages over linear synthetic reactions, viz., fewer steps, no purification of reaction intermediates (fewer purification steps), ease of automation, and the creation of a bioactive compound library in a short time. Notably, these MCRs have great potential in Medicinal Chemistry in order to establish lead compounds in a short period of time as well as on an industrial scale. The Westermann group [1] has employed MCRs, in particular the Ugi-four component reaction (U-4CR), to prepare semi-synthetic analogs of triterpene acids (betulinic acid and fusidic acid), and steroids (cholic acid) conjugated with TEMPO (nitroxide). Notably, the nitroxide labelled betulinic and fusidic acid derivatives illustrated much better cytotoxic effects on prostate cancer (PC3) and colon cancer (HT29). The authors investigated the mechanism of the active molecules, which showed that these semi-synthetic compounds increased the level of caspase-3 significantly, which indicated the induction of apoptosis by activation of the caspase pathway.

Sesquiterpene lactones are a group of natural products reported from numerous plant species and are abundant in the Asteraceae family. These molecules possess secondary metabolites with pharmaceutical applications for cancer therapy. Sesquiterpene lactones illustrated numerous fascinating biological activities viz., antiamoebic, trypanocidal, antigiardial, antibacterial, antidiabetic, antitumor, cytotoxic, and phytotoxic. The García-Hernández group [2] isolated the sesquiterpene lactone, incomptine A (IA) from *Decachaeta incompta*. Cytotoxic studies revealed that IA illustrates potent activity toward lymphoma cancer (U-937). The mechanistic investigation demonstrated that IA significantly enhances intracellular ROS levels along with the apoptotic activity. Moreover, in the proteomic investigation, 1548 proteins were differentially expressed. Of these 1548 proteins, 961 possessed a fold-change ≤ 0.67 and 587 possessed a fold-change ≥ 1.5. Notably, the majority of these proteins are involved in oxidative stress, apoptosis, and glycolytic metabolism.

Hepatocellular carcinoma (HCC) is a primary liver cancer that has high mortality and incidence worldwide. Furthermore, chemotherapeutic resistance is the major obstacle in HCC treatment. Violacein is an alkaloid featuring two indole units coupled via a pyrrolidinone ring. This alkaloid is produced by various bacterial strains, such as *Chromobacterium violaceum*, and demonstrates various biological effects, including anticancer effects. Kim et al. [3] revealed that violacein inhibits the stemness and proliferation of Hep3B and Huh7 HCC cells. Further study revealed that this molecule inhibited the proliferation of Hep3B and Huh7 HCC cells via inducing cell cycle arrest at the sub-G1 phase along with apoptotic cell death induction. Additionally, violacein induced MMP, enhanced ROS, upregulated p21 and p53, and activated the caspase (caspase-3, caspase-9) and PARP along with downregulation of ERK1/2 and AKT signalling. Violacein reduced the expression of CD133, Nanog, Oct4, and Sox2, via inhibiting STAT3/AKT/ERK pathways.

Solanidine and demissidine are leading compounds of the class “solanidane alkaloids” mainly found in the glycoside form in potato species, such as *Solanum tuberosum*, *S. acaule*, and *S. demissum*. Some members of the solanidane alkaloids illustrate potent antiproliferation properties and induce apoptosis in liver, cervical, stomach, and lymphoma cancer cells. Notably, the cytotoxic effects of α-chaconine toward HepG2 cells (hepatocellular cancer) are higher than the standards—camptothecin and doxorubicin. Wojtkielewicz et al. [4] established an approach for the synthesis of solanidanes from the spirostane sapogenin tigogenin. Moreover, the indolizidine core within the solanidane alkaloids was assembled in five steps starting from spirostane. In addition, numerous demissidine derivatives have been prepared from the alkaloid tigogenin through various imine intermediates.

Polygodial, a dialdehyde sesquiterpenoid, was reported from *Tasmannia lanceolata*. The literature revealed that ophiobolin A demonstrates potent effects toward apoptosis-resistant glioblastoma cells via the induction of a non-apoptotic cell death pathway. Maslivetc et al. [5] prepared dimers and trimers of polygodial and aphiobolin A in order to discover any metabolites that could crosslink biological primary amine-containing targets. The authors demonstrated that such molecules keep the pyrrolylation ability and illustrate increased single-digit micromolar potencies against apoptosis-resistant cancer cells.

The Macabeo research group [6] investigated the rice culture of *Sparticola junci* and produced the naphthoketal-bearing polyketides, sparticatechol A, sparticolin H, and sparticolin A. The absolute configurations of these natural products were established using ECD together with TDDFT. All three of these secondary metabolites proved to be highly active toward COX-2 and COX-1 enzymes, with sparticatechol A possessing the highest effects, which were higher than the standard Celecoxib. Furthermore, this molecule possesses preferential binding towards COX-2. Sparticolins H and A demonstrated moderate cytotoxic effects toward K-562 cells (myelogenous leukemia) and weak cytotoxicity toward mouse fibroblast cells (HeLa).

The genus *Sepedonium* is a tremendous source of natural products with intriguing chemical diversity. Nobert and his co-workers [7] investigated *Sepedonium* ampullosporum (strain KSH534) and isolated two new peptaibols, ampullosporin F and ampullosporin G, along with five known natural products. The structures of these molecules were established via extensive spectroscopic techniques. Moreover, the total synthesis of ampullosporins F and G was accomplished via a solid-phase strategy in order to establish the absolute configuration of all the chiral amino acids. Additionally, these two molecules possessed potent antifungal effects towards *Phytophthora infestans* and *Botrytis cinerea*. Furthermore, ampullosporins F and G demonstrated potent anticancer effects toward cell viability assays on PC-3 (prostate cancer) and HT-29 (colorectal cancer).

Microbial co-cultivation is a fascinating strategy employed in order to activate biosynthetic gene clusters. Furthermore, based on a comparative metabolomics approach and the anti-phytopathogenic effects of the co-cultures, Oppong-Danquah et al. [8] investigated *Magnaporthe oryzae* and the marine *Cosmospora* sp. Phytochemical investigation of *M. oryzae* and *Cosmospora* sp. produced five isochromanones, soudanones A, E, D, H, and I, along with the isochromans, pseudoanguillosporins A and B, naphtho-γ-pyrones, ustilaginoidin G, and cephalochromin. The basic structures of these compounds were established via NMR and absolute configuration through ECD together with Mosher’s ester reaction. Among the tested compounds, only soudanones E and D illustrated antimicrobial effects towards *Phytophthora infestans* and *M. oryzae*, while pseudoanguillosporin A possessed potent anti-phytopathogenic effects towards *Xanthomonas campestris*, *Pseudomonas syringae*, *P. infestans*, and *M. oryzae*.

Despite the remarkable advances in immuno- and targeted therapies, breast and lung cancer are among the leading causes of cancer death. Combination therapy is an innovative strategy where a mixture of different drugs is used to treat diseases. Sulaiman et al. [9] explored such a combination therapy by using butein together with an anticancer flavonoid on lung (A549) and breast cancer (MDA-MB-231) cells together with another anticancer triterpene, frondoside-A. The authors demonstrated that butein was able to reduce the two cancer cell colony’s growth and viability. In addition, this combination therapy decreases tumour growth on the chick embryo chorioallantoic membrane (CAM) in vivo. The authors further demonstrated that the anti-cancer effects of butein are due to significant inhibition of STAT3 phosphorylation, leading to PARP cleavage. This combination therapy was found to lead to synergistic effects on the inhibition of HUVEC migration.

Target identification is a challenging and important strategy for identifying drug lead development. In this regard, Src tyrosine kinase has been extensively developed as a factor in tumorigenesis via differentiation, regulating cell growth, survival, and adhesion. Wu et al. [10] developed a novel in silico docking strategy for the target identification of kaempferol. These results were further validated via TargetHunter and PharmMapper server protocols. From computational studies, it has been confirmed that Src is a validated target for kaempferol, and this validation was additionally verified by kaempferol cardioprotective potential in vitro and in vivo screening.

Cervical cancer is the fourth most common cancer in women and a leading cause of death for women in the US. The steroidal saponin, RCE-4 [(1β, 3β, 5β, 25*S*)-spirostan-1,3-diol-1-[α-L-rhamnopyranosyl-(1→2)-β-D-xylopyranoside] is produced by *Reineckia carnea*. The Chen research group [11] investigated the mode of action of RCE-4 towards cervical cancer and targeted the Bcl-2–Beclin 1 complex, an essential programmed cell death (PCD) regulator. Notably, the results of the Chen group illustrate that this molecule inhibited the formation of the Bcl-2–Beclin 1 complex and that the ATG 4B proteins served as a crucial co-factor. In addition, the sensitivity of RCE-4 to Ca Ski cells was increased remarkably by inhibiting the expression of the ATG 4B.

Melanoma is one of the most heterogeneous and aggressive cancers possessing a strong capability to evolve resistance towards different therapeutic strategies. Melanoma remains a most challenging cancer to treat because of foetal safety and balancing maternal needs. Schrom et al. [12] cultured four lymph node metastasis-derived pigmented and non-pigmented sections. Furthermore, the four cultures possessed different genotypic, phenotypic, and tumorigenic properties. For the treatment protocol, synthetic human lactoferricin-derived peptides were screened. Based on their scientific data, the authors claimed that these anti-tumour peptides could be employed as an optional alternative innovative therapeutic treatment during pregnancy.

Lung cancer also remains one of the leading cancer-related deaths that continues to challenge researchers involved in searching for lead compounds to treat this cancer. The tumour microenvironment (TME) is considered one of the leading signs of epithelial cancers, such as most lung cancers, and is associated with progression, tumorigenesis, metastasis, and invasion. Yang et al. [13] published a review that emphasised the important part of the TME and comprehensively described the antitumour properties and mode of action of numerous natural products that target the TME. Furthermore, the authors discussed combination therapy, i.e., the synergistic properties of various natural products employed in combination with other anticancer molecules. The authors also emphasised the use of nanotechnology previously used to increase the anticancer potentials of natural products.

Cancer stem cells (CSCs) are crucial factors for tumour stemness by increasing proliferation, colony formation along with metastasis. Furthermore, CSCs may also be employed as a potential therapy resistance protocol. The presence of CSCs can cause cancer recurrence, and their complete elimination can have significant therapeutic benefits. The review of Paskeh et al. [14] emphasises natural products-based targeting CSCs in cancer therapy. Numerous dietary aspects have been covered in this review on CSCs, including flavones, flavonols, chalcones, isoflavones, caffeic acid, cartenoids, and ginsenosides. Various molecular pathways such as the Sonic Hedgehog, Wnt/β-catenin, STAT3, NF-κB, and Gli1 that follow these molecules in suppressing CSC are also featured. The authors reported that upon exposure to these natural products, a potential decrease occurs in the CSC markers’ levels, such as CD133, CD44, Oct4, and ALDH1, in order to impair cancer stemness.

The ongoing COVID-19 pandemic situation created by SARS-CoV-2 has become a leading health issue globally over the past two years. Kaul et al. [15] published a review that focussed on research data from in vitro screening of flavonoids on key SARS-CoV-2 targets. They analysed 27 research papers that included over 69 flavones for their anti-SARS-CoV-2 targets. The combination of flavonoids with other synthetic drugs demonstrated promising results. They further emphasised the importance of in silico studies of flavonoids towards SARS-CoV-2 and highlighted the clinical studies. The authors claimed that flavonols, myricetin, quercetin, baicalein, baicalin, and the flavan-3-ol EGCG, along with tannic acid, have great potential for in vivo evaluation and clinical studies.

Bispecific antibodies (bsAbs) were developed in the 1960s and are now considered to be a leading group of immunotherapies to treat cancer. Furthermore, numerous different bsAbs have been documented in the last decade, mainly generated genetically. Bordusa et al. [16] published a novel chemo-enzymatic protocol for generating bsAbs fragments through the covalent fusion of two functional antibody Fabs. They initially modified the single Fabs site through click anchors employing an enhanced Trypsiligase variant (eTl), and this was later followed by conversion into the heterodimers through click chemistry. In later stages, the authors employed the inverse electron-demand Diels–Alder reaction and strain-promoted alkyne-azide cycloaddition protocols, which are well-established strategies for their diminished side reactions and fast reaction kinetics. The authors also developed enzymatic C-C and C-N terminal coupling protocols of the two Fabs through peptide linkages. The resulting bsFabs illustrate cytotoxic effects on breast cancer cells.
